# Unraveling the pathways of sustainable music education: a moderated mediation analysis of environmental awareness, pedagogical approaches, and student engagement

**DOI:** 10.3389/fpsyg.2025.1554944

**Published:** 2025-05-09

**Authors:** Kun Luo, Yi Wang

**Affiliations:** College of Music, Guangdong Polytechnic Normal University, Guangzhou, China

**Keywords:** sustainable music education, environmental awareness, innovative pedagogical approaches, student engagement in music learning, moderated mediation analysis

## Abstract

**Introduction:**

Given the significance of fostering sustainable practices in music education, the study proposes a relation between sustainable music education (SME) and environmental awareness (EA) in music learning through a moderated mediation mechanism of student engagement (SE) and innovative pedagogical approaches (IPA).

**Methods:**

The study employs a survey-based approach to empirically assess the hypothesized model. Data are collected through questionnaires from a sample of music students in China. Further, data are analyzed using multivariate analytical techniques to establish relationships among these variables.

**Results:**

Our findings indicate that SME exhibits a significant influence on EA and consciousness among music students. Besides, SE significantly mediates the link between SME and EA. Furthermore, IPA moderates the association between SME and EA through the mediator effect of SE, such that at high levels of IPA, the associations are stronger (vice versa).

**Discussion:**

This study contributes to the field of music education by unveiling the pathways through which SME can foster heightened EA. By establishing a moderated mediation model involving SE and IPA, this research highlights the interconnectedness of these variables in the context of music learning.

## Introduction

Environmental awareness (EA) refers to “the acknowledgment, comprehension, and apprehension about the environment, which encompasses ecological systems, natural resources, and the consequences of human actions on the planet” (Galli et al., 2020; Niu et al., 2022). The concept involves recognizing the interdependence between human activities and the natural environment, as well as understanding the possible ramifications of these activities, encompassing concerns such as pollution, climate change, and habitat deterioration (Oe et al., 2022). According to Niu et al. (2022), the scope of EA expands beyond mere recognition to include a comprehensive understanding of sustainable methodologies and a profound commitment to safeguarding and sustaining the natural environment. In the field of music education, EA pertains to the comprehension of the ecological consequences associated with musical activities, as well as the adoption of sustainable decisions in creative pursuits (Brennan et al., 2019; Oe et al., 2022; Weng and Chen, 2020).

The significance of cultivating EA among music students is rooted in the distinct societal role that musicians assume. Artists possess the capacity to engage a wide range of individuals and effectively convey significant themes through their artistic creations. In a recent study conducted by Bartleet et al. (2019), it was found that music has the capacity to effectively communicate environmental issues and motivate individuals to take action. According to Browne et al. (2019), the author aims to foster EA among music students, enabling them to not only make environmentally conscious decisions in their musical pursuits but also to actively promote environmental sustainability. The significance of this matter is progressively crucial within a global context characterized by urgent environmental predicaments. Nevertheless, there are still areas of research that need to be explored in order to have a better knowledge of the development of EA among music students (Solomos and Higgins, 2023). Although there is an increasing amount of scholarly work on environmental education, there is a scarcity of research that particularly examine the factors that influence students' environmental attitudes within the realm of music education. The existence of this gap poses a challenge to our capacity to develop efficient educational interventions that are specifically catered to the distinct requirements of music students.

From this standpoint, the concept of sustainable music education (SME) arises as a potentially effective strategy to bridge this disparity. SME refers to the integration of sustainability principles into music teaching and learning, encompassing environmental, social, and economic dimensions (Brennan et al., 2019; Park, 2022). It promotes ecological consciousness, ethical music practices, and long-term preservation of musical heritage while minimizing environmental impact. SME encourages students to critically assess the ecological footprint of musical activities, such as instrument production, concert energy consumption, and material waste, fostering responsible decision-making in their artistic endeavors (Guo et al., 2020; Weng and Chen, 2020).

According to Brennan et al. (2019), the integration of sustainability principles into music education by SME involves a focus on the environmental and social consequences of musical practices. Similarly, Park (2022) and Smith (2022) highlight the importance of SME in promoting students' comprehension of environmental matters and fostering their inclination toward adopting environmentally conscientious actions. In a similar vein, Yu et al. (2023) contend that SME presents a comprehensive framework that fosters the development of critical thinking skills pertaining to the environmental impacts associated with the production and performance of music. Putatively, the extant body of scholarly work pertaining to SME posits the necessity for more investigation and underscores the significance of incorporating sustainability principles into music education. This integration aims to foster the development of environmentally aware musicians who can actively contribute to the advancement of a more sustainable future.

In addition, the study investigates the relationship between SME and EA, with a focus on understanding how student engagement (SE) acts as a mediating variable in this linkage. Building on Bandura's social cognitive theory (Bandura, 1989), which highlights the intertwined nature of learning and behavior, the study posits that SME influences SE, as actively engaged students are more likely to embrace sustainable music practices (Barrón et al., 2022). Moreover, the author submits that this heightened SE, in turn, mediates the pathway between SME and EA, implying that engaged students are more inclined to internalize and apply sustainability principles (Sökmen, 2021), ultimately leading to greater EA. This research seeks to empirically examine and provide insights into the dynamic relationship between these constructs, shedding light on the pivotal role played by student engagement in the journey from SME to enhanced EA.

**Figure 1:**
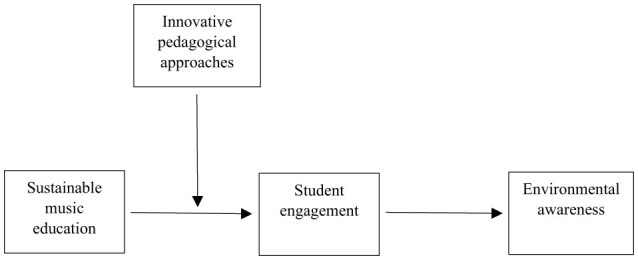
Conceptual model.

Furthermore, this study extends the current research by assessing innovative pedagogical approaches (IPA) as a contextual factor that may influence these relationships. IPA encompasses dynamic teaching methodologies designed to enhance learning experiences, including strategies like project-based learning and technology integration (Konst and Kairisto-Mertanen, 2020). The rationale for incorporating IPA as a moderator lies in the diversity of pedagogical approaches within music education, suggesting that the effects of SME on SE and SE's subsequent mediation of the relationship between SME and EA may vary based on the specific pedagogical approach utilized. Furthermore, IPA inherently promote active SE (Sharoff, 2019). Given that SE is proposed as a mediator, IPA's potential to stimulate deeper and more sustained engagement among students may enhance the mediation effect. By including IPA as a moderator, the study aims to investigate how the degree and nature of pedagogical innovation interact with the proposed relationships. This approach allows us to gain insights into the nuanced dynamics of sustainable music education and its educational outcomes. The moderated mediation model is depicted in Figure 1.

## Literature review

### Sustainable education and its role in music learning

Sustainable education is an evolving pedagogical framework that emphasizes ecological responsibility, social equity, and economic sustainability in learning environments (Tripon et al., 2023). It aims to cultivate students' awareness of global sustainability challenges while equipping them with the knowledge, values, and skills to contribute positively to their communities (Felgendreher and Löfgren, 2018). By integrating sustainability themes into curricula, educational institutions foster a mindset that encourages critical thinking, problem-solving, and ethical decision-making—skills essential for addressing contemporary environmental concerns (Østergaard, 2019).

Within the context of music education, sustainability extends beyond theoretical knowledge to include experiential learning, eco-friendly performance practices, and community engagement. Music programs that adopt sustainability principles often incorporate interdisciplinary approaches, blending music with environmental sciences, ethics, and technology to enhance student awareness (Yu et al., 2023). For instance, students may engage in projects that explore the lifecycle of musical instruments, evaluate the carbon footprint of live performances, or create compositions that reflect environmental themes (Bartleet et al., 2019).

Several initiatives worldwide illustrate how sustainable music education can be integrated into curricula and music practice. Eco-friendly instrument production has gained attention, with students learning to create and use instruments made from repurposed materials such as wood from fallen trees or recycled metals (Guo et al., 2020). Similarly, many institutions are transitioning to digital and paperless sheet music, reducing paper waste and improving accessibility (Park, 2022). Advances in technology also support green concert initiatives, where energy-efficient stage lighting, carbon-neutral performances, and solar-powered recording studios are implemented to minimize environmental impact (Brennan et al., 2019). These practices not only promote environmental consciousness among students but also encourage the development of sustainable habits that extend beyond the classroom and into their professional music careers.

### Hypotheses

SME is an emerging field of study focused on examining the complex environmental and societal effects inherent in various musical activities (Skjerven and Fordham, 2022). The scholarly investigation, as elucidated by Mariguddi (2023) and Weng and Chen (2020), positions SME as a central focus in the context of music education. According to Skjerven and Fordham (2022), the integration of sustainability topics in music education by SME offers a distinctive and captivating approach to familiarizing students with environmental concerns. Similarly, Cheng (2015) argued for the incorporation of sustainability themes within the music curriculum, postulating that this approach establishes a direct connection between music and the environment, thereby encouraging students to actively engage with these concerns. Furthermore, the emotive characteristics of music have the ability to elicit intense feelings and foster empathy (Dewaele, 2019). Consequently, this emotional attachment to environmental topics in music can result in increased consciousness and apprehension toward ecological matters. This phenomenon can be attributed to the fact that individuals are more inclined to engage in proactive behavior when they experience a strong emotional attachment to a particular cause. Furthermore, SME frequently incorporates pragmatic initiatives pertaining to sustainability, shown by the utilization of recycled materials for musical instruments or the implementation of energy-saving measures during music productions (Guo et al., 2020). Therefore, the implementation of these sustainable practices in the realm of music education has the potential to foster a heightened consciousness of sustainable behaviors in daily life (Prior, 2022). Therefore,

H1. Sustainable music education will positively influence students' environmental awareness.

In the context of music education, SE plays a pivotal role in shaping EA, and this relationship can be effectively understood through the lens of SCT (Bandura, 1989). SCT posits that individuals learn and develop their beliefs, attitudes, and behaviors through a complex interplay of personal factors, environmental influences, and behavioral factors. Applying SCT to the context of music education allows us to explore how engaged music students become more environmentally aware. Firstly, SCT highlights the importance of observational learning (Bandura, 2023). According to Chen and O'Neill (2020), engaged music students are more likely to actively participate in environmentally conscious musical activities and observe the behaviors and attitudes of their peers and instructors. For instance, when music students engage in discussions about the environmental impact of music production or collaborate on sustainable music projects, they observe others who are passionate about environmental issues. Subsequently, this observation fosters a sense of community and inspiration, promoting greater EA (Lee et al., 2019). Secondly, SCT engenders the role of self-efficacy in shaping behavior (Bandura, 2023). This argument is substantiated by Maricuţoiu and Sulea (2019), who endorsed that engaged music students tend to have higher self-efficacy when it comes to making environmentally responsible choices within the realm of music. Their active involvement in sustainable music practices, such as using eco-friendly instruments or advocating for green concert venues, contributes to their belief in their capacity to make a positive environmental impact (Piyapong, 2020). Furthermore, the social context of music education is vital in SCT. Elevated levels of SE leverage them to collaborate with peers, participate in group performances, and engage in discussions about the role of music in society (Rabun et al., 2020). These interactions provide a platform for the exchange of ideas, information, and perspectives on environmental awareness within the musical context. Accordingly, through dialogues with fellow students and music instructors, students may be exposed to diverse viewpoints and develop a deeper understanding of the intersection between music and environmental sustainability (Yusuf and Fajri, 2022). Another tenet of SCT reinforces self-regulation and goal setting (Bandura, 1989), which enable students to set goals related to environmental sustainability in their musical pursuits. They may aim to reduce the carbon footprint of their musical performances or strive to incorporate more eco-conscious practices into their music education. By monitoring their progress toward these goals and adjusting their musical behaviors accordingly, they can develop a sustained commitment to environmentally responsible music practices (Alsaati et al., 2020). Therefore,

H2. Student engagement will positively influence students' environmental awareness.

According to SCT, SE acts as a crucial mediator that plays a pivotal role in bridging the pathway between SME and heightened EA. SME, with its emphasis on sustainability principles and practices within music education, serves as the catalyst that triggers the process (Varkøy and Rinholm, 2020). When students actively engage with SME initiatives, they become more immersed in their music learning experiences, which in turn fosters a deepened connection to the environmental and social implications of their musical practices (Sökmen, 2021). This heightened SE, driven by SME, contributes to students' cognitive and emotional involvement with sustainability matters. They not only acquire knowledge related to environmental concerns but also form a strong emotional attachment to these issues, driven by the emotive characteristics of music (Dewaele, 2019). This emotional engagement further fuels their commitment to understanding and addressing ecological perspectives. Consequently, the mediation effect of SE facilitates the internalization and application of sustainability principles in their daily lives, leading to a profound increase in environmental awareness (Salta et al., 2022). Therefore,

H3. Student engagement mediates the association between sustainable music education and environmental awareness.

IPA in the context of SME represent a dynamic and progressive approach to teaching sustainability concepts within music education. These pedagogical approaches encompass a wide range of methods and strategies that go beyond traditional teaching practices (Espeland, 2021). Examples of IPA in SME might include project-based learning initiatives where students create environmentally-themed musical compositions or experiential learning opportunities such as field trips to eco-friendly concert venues (Green, 2012). The underlying premise of IPA is to foster deep engagement and critical thinking among music students, encouraging them to explore sustainability issues and their connection to music in a more profound and transformative manner.

The study predicts that IPA influence the strength and nature of the link between SME and SE. As innovative pedagogical approaches are implemented, they can enhance the level of engagement experienced by music students. IPA can stimulate curiosity, creativity, and motivation, making sustainability concepts more accessible and compelling (Cebrián et al., 2020). For instance, incorporating cutting-edge technologies, interactive online platforms, or gamification elements into music lessons can captivate students' interest and encourage them to explore sustainability in novel and engaging ways. Furthermore, IPA can tailor the learning experience to the specific needs and interests of music students, which in turn can amplify the moderating effect. For instance, using technology-driven approaches such as virtual reality simulations to explore sustainable concert venue design can captivate the interest of students who are technologically inclined, thus increasing their SE (Lozano et al., 2017). In the similar vein, incorporating elements of cultural diversity and social justice within sustainability-focused music lessons can resonate with students who are passionate about social issues. By adapting IPA to align with the diverse interests and learning styles of music students, instructors can optimize the moderating role of IPA in bridging SME and SE. Moreover, IPA can foster a sense of ownership and agency among music students, which is instrumental in enhancing SE. When students are given the autonomy to choose sustainability topics that align with their interests and values, they become more personally invested in the learning process (Carey et al., 2018). Thus, IPA moderates the relationship between SME and SE by amplifying the impact of SME on SE, ultimately resulting in more profound environmental awareness and sustainable behaviors among music students. Therefore,

H4. Innovative pedagogical approaches moderate the link between sustainable music education and student engagement, such that at higher levels of innovative pedagogical approaches, the association between sustainable music education and student engagement becomes stronger.

Marrying these two assumptions (H3 and H4)—the influence of SME on EA through SE and the moderating role of IPA between SME and SE—it is plausible to suggest the moderated mediating role of IPA into the relationship between SME and EA through the mediator effect of SE. Therefore,

H5. Innovative pedagogical approaches moderate the link between sustainable music education and student engagement, such that at higher levels of innovative pedagogical approaches, the association between sustainable music education and environmental awareness through student engagement becomes stronger.

## Methodology

### Research objectives

This study aims to examine the role of SME in fostering EA among music students through a moderated mediation model. Specifically, the study investigates the direct relationship between SME and EA, the mediating role of SE in this link, and the moderating role of IPA in influencing the strength of these relationships. Additionally, the study explores the overall moderated mediation effect of IPA in shaping the relationship between SME, SE, and EA. To empirically validate these relationships, a quantitative research approach was adopted, utilizing a survey-based methodology.

### Research design and sampling

The current investigation employed a time-lagged research methodology to systematically collect data from a sample of university students in China who are actively pursuing a music degree. The decision to implement a time-lagged approach was driven by the need to minimize common method bias and enhance the validity of the findings. Unlike traditional cross-sectional designs that capture data at a single time point, this method allowed for a structured, phased collection of responses, reducing the risk of response bias and providing a more reliable assessment of causal relationships among the study variables. Given the complexity of investigating sustainable music education, environmental awareness, student engagement, and innovative pedagogical approaches, this approach ensured that participant responses were not influenced by immediate situational factors but rather reflected their genuine experiences and perceptions over time.

To ensure targeted and purposeful participation, a purposive sampling technique was adopted. The study specifically focused on students who were actively engaged in music education programs across multiple institutions in China, ensuring that participants had direct exposure to sustainability concepts in music learning. This targeted approach was crucial in obtaining relevant insights from individuals who could meaningfully reflect on how sustainability principles were integrated into their academic and creative pursuits. Participation was entirely voluntary, and students were provided with detailed information about the study's objectives before giving their consent. To encourage participation and ensure informed responses, the survey was distributed both digitally and in print, and multiple follow-ups were conducted via email and institutional communication platforms. Participants were also assured that they had the right to withdraw at any stage without any consequences.

Data collection took place over a 4-month period (February 2023–May 2023) and was executed in three distinct phases to align with the time-lagged structure. In the first wave, a total of 500 questionnaires were distributed to collect baseline data on SME, EA, and demographic details. Of these, 446 responses were received, but 23 were excluded due to incomplete or inconsistent information, leaving a valid sample of 423 participants. The second wave focused on gathering responses related to SE, using the same participants who completed the first phase. In this phase, 403 valid responses were collected, indicating a high retention rate across phases. The third and final wave involved the assessment of IPA, with responses collected from the remaining participants.

To maintain data integrity across these multiple collection points, a unique identification system was implemented. Participants were instructed to generate a secure but anonymous key based on a combination of their zip code and initials, allowing responses to be accurately matched across the three waves while preserving confidentiality. This approach significantly reduced the likelihood of mismatched responses and enhanced the reliability of longitudinal comparisons. After the final data consolidation, the study yielded a final sample of 392 complete responses, which were used for analysis.

The demographic profile of the sample provided a well-balanced representation of students at different academic levels. The average age of the participants was 34.39 years (SD: 5.87). In terms of gender distribution, 44% of the students identified as male, while 56% identified as female. Regarding academic levels, 33% were enrolled in undergraduate programs, 38% in graduate programs, and 29% in postgraduate programs. This diversity in academic standing allowed the study to assess how sustainability concepts in music education are perceived and internalized at different stages of academic training.

### Measures

The researcher modified the research instruments adapted from previous studies to gather data from the intended participants in order to capture the anticipated links. The survey was administered in the Chinese language using a back-translation method, as suggested by Brislin (1980). The scale questions were assessed using a Five-point Likert scale, with responses ranging from 1 (strongly disagree) to 5 (strongly agree). Each construct was measured using validated multi-item scales adapted from previous research.

To ensure content validity, minor modifications were made to the wording of some items to align with the context of music education and sustainability. Additionally, participants were asked to respond based on their personal experiences, perceptions, and educational exposure to sustainable music practices.

### Sustainable music education

The instrument to assess SME was developed from previous studies (e.g., Guo et al., 2020; Jian, 2022; Park, 2022). The instrument consists of four items. The survey included statements such as “My music education includes discussions about sustainability and its relevance to the music industry” and “I have opportunities to engage in sustainable music practices, such as using eco-friendly instruments.” Additionally, other items assessed whether instructors encouraged the use of digital sheet music to reduce paper waste and whether sustainability concepts were incorporated into coursework and assignments.

### Environmental awareness

To measure environmental awareness (EA), a four-item scale adapted from Noordin and Sulaiman (2010) was used. The questions focused on students' knowledge, concern, and actions regarding sustainability. Participants responded to statements such as “I deliver information about environmental sustainability to my family and peers” and “I actively participate in discussions on the environmental impact of music production.” Other items examined whether students considered environmental factors when making decisions about musical activities and their awareness of sustainable practices in music education.

### Student engagement

Student engagement (SE) was assessed using Cadime et al.'s (2016) Utrecht Engagement Scale, which includes 17 items measuring cognitive, emotional, and behavioral engagement in learning. The survey asked participants to rate their agreement with statements such as “In my studies, I feel like I am bursting with energy” and “Time flies when I'm studying music-related topics.” Additional items measured the extent to which students engaged in sustainability discussions within their music courses and whether they actively sought opportunities to integrate sustainability into their musical learning and practice.

### Innovative pedagogical approaches

To evaluate innovative pedagogical approaches (IPA), a ten-item scale adapted from Akdeniz et al. (2019), Santos et al. (2019), and Wu and Chen (2021) was used. This scale assessed the role of creative teaching methodologies in promoting sustainability in music education. Participants responded to items such as “The innovative pedagogical approaches in my music education allow me to apply sustainability principles practically in musical projects and performances” and “My instructors incorporate technology and digital tools to enhance sustainable learning in music education.” The survey also examined whether project-based learning activities related to sustainability were integrated into music courses and whether teachers encouraged collaboration and creativity in sustainability-focused music education.

### Data analysis plan

The collected data were analyzed using variance-based structural equation modeling through SmartPLS (version 4.0). A two-step approach was adopted, beginning with an assessment of the outer measurement model to establish the reliability and validity of constructs, followed by an examination of the inner structural model to test the hypothesized relationships. To ensure robustness, reliability was assessed using Cronbach's alpha and composite reliability, while validity was evaluated through convergent and discriminant validity tests. Hypothesis testing was conducted using bias-corrected and accelerated bootstrapping with 3,000 resamples to derive path coefficients and significance levels. Additionally, moderation and mediation analyses were performed to examine the interaction effects of innovative pedagogical approaches on the relationship between SME, SE, and EA.

## Results

The researcher employed variance-based structural equation modeling (SEM) to examine the postulated associations between variables using SmartPLS (version 4). The reflecting model was subjected to analysis using the Partial Least Squares Structural Equation Modeling (PLS-SEM) technique, as described by Ringle et al. (2022). To investigate the proposed relationships, the study initially analyzed the outer model. Following the successful validation of the outer model, the inner model was next evaluated to determine the structural routes. Comprehensive analysis is provided in the following subsections.

### Outer model assessment

The outer model was evaluated by the author in accordance with the recommendations of Hair et al. (2018) in order to establish the reliability and validity of the constructs. The reflective measurement model was assessed using SmartPLS (v 4.0), and various tests were conducted to validate the outer model. These tests included the evaluation of reliability using metrics such as Cronbach's alpha and composite reliability (CR). Convergent validity was determined by examining outer loadings and the average variance extracted (AVE) criteria. Discriminant validity was established through the Fornell-Larcker and heterotrait-monotrait (HTMT) ratio, as recommended by Hair et al. (2018). The findings of the internal consistency and reliability study are displayed in Table 1. The results indicate that the Cronbach's alpha and CR values are within the acceptable range of 0.70 and 0.95 (Cronbach, 1951). Moreover, it is worth noting that the outer loadings and average variance extracted (AVE) values beyond the permissible threshold of 0.50, as established by Henseler et al. (2015). The aforementioned findings provide support for the reliability and convergent validity of the study tools.

**Table 1:** Assessment of reliability and validity of constructs.

**Items**	**Constructs**	**α/CR**	**AVE**	**Factor loading**
SME	Sustainable music education	0.895/0.921	0.713	
SME1				0.813
SME2				0.745
SME3				0.927
SME4				0.884
EA	Environmental awareness	0.867/0.891	0.701	
EA1				0.858
EA2				0.866
EA3				0.762
EA4				0.858
SE	Student engagement	0.863/0.890	0.604	
SE1				0.716
SE2				0.893
SE3				0.714
SE4				0.783
SE5				0.801
SE6				0.823
SE7				0.781
SE8				0.754
SE9				0.729
SE10				0.684
SE11				0.790
SE12				0.699
SE13				0.844
SE14				0.892
SE15				0.748
SE17				0.748
IPA	Innovative pedagogical approaches	0.838/0.867	0.638	
IPA1				0.777
IPA2				0.715
IPA3				0.859
IPA4				0.904
IPA5				0.821
IPA6				0.711
IPA7				0.729
IPA8				0.837
IPA9				0.727
IPA10				0.880

Following the successful validation of convergent validity, the author proceeded to assess the discriminant validity through the utilization of the Fornell-Larcker criterion and the HTMT ratio. The values presented in Table 2, known as the Fornell-Larcker values, are derived from the square root of the AVE. These values serve as an indicator of the strength of inter-construct correlations compared to outer-construct correlations (Hair et al., 2018). Additionally, Table 3 displays the HTMT values that were produced by the utilization of the bias-corrected and accelerated (BCa) bootstrapping technique, with a total of 3,000 iterations. The researcher adhered to the guidelines proposed by Henseler et al. (2015) and evaluated the HTMT ratio with a one-tailed test at a significance level of 90%. Through this process, the author was able to derive the appropriate confidence intervals (CIs) with a significance level of 95% (two-tailed). The findings suggest that all the observed values fall below the established threshold of HTMT 0.90, indicating their compliance with the acceptable level.

**Table 2:** Assessment of Fornell-Larcker.

**Constructs**	**SME**	**EA**	**SE**	**IPA**
SME	0.844			
EA	0.525	0.836		
SE	0.633	0.515	0.777	
IPA	0.361	0.612	0.405	0.798

**Table 3:** Assessment of HTMT ratio.

**Constructs**	**SME**	**EA**	**SE**	**IPA**
SME				
EA	0.632			
SE	0.542	0.541		
IPA	0.725	0.619	0.636	

### Inner model assessment

The researchers investigated the structural model: inner model to derive path coefficients (β), coefficient of determination (*R*^2^), predictive relevance (*Q*^2^), and effect size (*f*^2^) after validating the outer model. The *t*- and *p*-values required for a 95% significance level were derived using the BCa bootstrapping method with 3,000 iterations (Hair et al., 2018). The results indicating a direct relationship between the variables under consideration are presented in Table 4.

**Table 4:** Assessment of structural model.

**Effects**	**Relationship**	**Path value**	***P* value**	***T* value**	***R^2^* value**	***F^2^* value**
Direct effects	SME → EA (H1)	0.447	0.000	8.762	0.496	0.283
	SE → EA (H2)	0.512	0.000	11.362	0.504	0.310
Indirect effect	SME → SE → EA (H3)	0.293	0.000	8.543		0.410
Moderation effects	SMExIPA → SE (H4)	0.233	0.003	2.693		0.212
	SMExIPA → SE → EA (H5)	0.344	0.000	4.813		0.174

To address objective 1, which seeks to examine the direct influence of SME on EA, Hypothesis H1 was tested. The findings indicate that SME has a significant and positive effect on EA, with a β value of 0.447 and a significant *t*-value of 8.762 (*p* < 0.05). This supports the assertion that integrating sustainability within music education contributes to heightened environmental consciousness among students.

Similarly, in line with objective 2, which examines the direct effect of SE on EA, Hypothesis H2 was tested. The results confirm that SE significantly influences EA, with a β value of 0.512 and a significant *t*-value of 11.362 (*p* < 0.05). The effect sizes for these relationships are 0.283 and 0.310, respectively, suggesting that both SME and SE exert a medium effect on EA.

To address objective 3, which investigates whether SE mediates the relationship between SME and EA, Hypothesis H3 was tested. The researchers employed BCa bootstrapping with 3,000 iterations to generate the indirect effects. The findings indicate that there is a statistically significant mediation effect, with a β value of 0.293 and a significant *t*-value of 8.543 (*p* < 0.05). These results suggest that SE plays a crucial role in transmitting the effects of SME onto EA. Moreover, the effect size (*f*^2^ = 0.410) is classified as large, reinforcing the importance of SE as a mediating factor in this relationship.

To address objective 4, which assesses whether IPA moderate the relationship between SME and SE, Hypothesis H4 was tested. The results presented in Table 4 indicate that the interaction effect between SME and IPA is statistically significant for SE, with a β value of 0.233, a *t*-value of 2.693, and *p* = 0.003. The effect size (*f*^2^ = 0.212) confirms that IPA strengthen the relationship between SME and SE, particularly when such approaches are implemented at high levels.

Furthermore, in addressing objective 5, which explores the moderated mediation effect of IPA in the relationship between SME and EA through SE, Hypothesis H5 was tested. The results demonstrate that at higher levels of IPA, the indirect effect of SME on EA through SE becomes significantly stronger (β = 0.344, *t* = 4.813, *p* = 0.000, *f*^2^ = 0.174). This confirms that pedagogical innovations in music education enhance both direct engagement and the subsequent development of EA among students.

**Figure 2:**
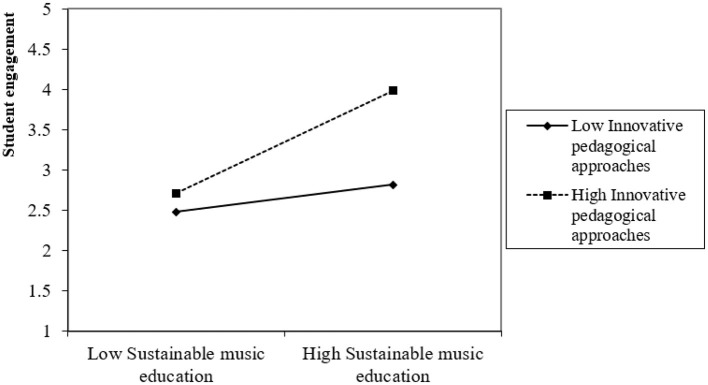
Moderation effect.

**Figure 3:**
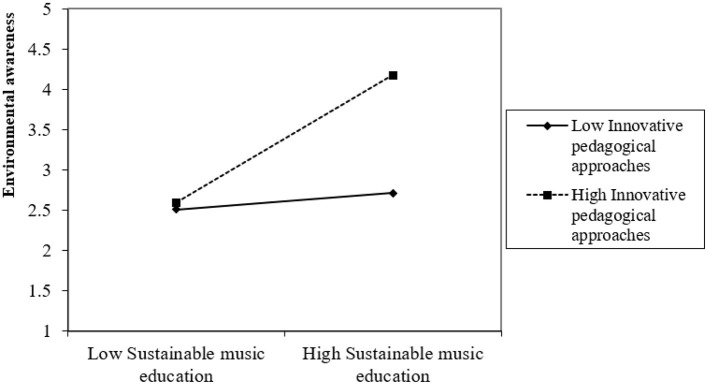
Moderated mediation effect.

In addition, the author examined the simple slope interaction effect of IPA on the association between SME and SE (as well as EA through SE). The graphical representation of the interaction effect is illustrated in Figures 2, 3. These figures provide support for the hypothesized associations by demonstrating that the direct relationship between SME and SE, as well as the indirect association between SME and EA through SE, are more pronounced at elevated levels of IPA. Conversely, the strength of these relationships diminishes at lower levels of IPA. Finally, in order to assess the goodness-of-fit (GOF) index, the author assessed the standardized root mean square residual (SRMR) and the analysis yielded the value of 0.057 < 0.08, signifying the goodness fit of the proposed model.

## Discussion and conclusion

The study delves into the relationship between SME, EA, SE, and IPA in the realm of music education. Our findings reinforce the significant influence of SME on EA, emphasizing its role in cultivating environmental consciousness among music students. This aligns with the growing recognition of the importance of sustainability in music education (Coss, 2013; Varkøy and Rinholm, 2020). The study projected that SME serves as a foundation upon which students can build a deeper understanding of the ecological impact of music-related activities. One of the notable contributions of this research lies in its exploration of the mediating role of SE. The results reveal that SE effectively mediates the link between SME and EA. Engaged music students, who actively participate in sustainability-themed music activities and internalize sustainability values, are more likely to develop a heightened environmental awareness. Furthermore, this study sheds light on the moderating effect of IPA. It highlights that IPA plays a crucial role in strengthening the relationship between SME and EA, facilitated through the mediator SE. When innovative pedagogical approaches are incorporated into music education, the association between SME and EA becomes more pronounced, particularly at higher levels of IPA. This suggests that the use of creative teaching methods and strategies intensifies the positive impact of SME on students' EA.

### Theoretical implications

The research presents significant implications for the advancement of theory. The study's findings provide substantial support for H1, which posited that SME will positively influence EA. While empirical data in this study supports the positive influence of SME on EA, this finding aligns with and extends previous research that has established the positive impact of sustainable education on the environmental behaviors of students. Firstly, the theoretical implications of our study resonate with the broader literature on sustainable education. Previous research has consistently demonstrated the effectiveness of sustainable education programs in enhancing students' environmental behaviors and attitudes (Felgendreher and Löfgren, 2018; Michelsen and Fischer, 2017). This study's findings specifically within the context of music education reinforce the idea that SME can serve as a catalyst for heightened environmental awareness. This aligns with the broader theoretical framework that suggests that education, when infused with sustainability principles, can empower students to become more environmentally conscious and responsible (Østergaard, 2019).

The second hypothesis H2 positing the positive influence of SE on EA is validated by empirical finding that yielded statistically significant connection among these variables. This empirical evidence reaffirms the theoretical framework underpinning the link between SE and EA and contributes to the broader understanding of how engagement can shape environmentally conscious behaviors. For instance, our study reinforces the theoretical premise that active engagement in sustainability-related activities and learning experiences can lead to heightened EA (Cogut et al., 2019). The study's findings support this theoretical perspective by showcasing that engaged students, who actively participate in sustainability-themed activities within their music education, tend to internalize sustainability values and develop a deeper understanding of environmental issues. Secondly, our research extends the theoretical understanding of how SE influences sustainability endeavors. Previous studies have highlighted the positive impact of SE on pro-environmental behaviors and sustainable practices in a broader educational context (Servant-Miklos et al., 2023). However, examining this relationship in the context of music education is unique and germane, which just makes this research all the more salient to offer theoretical as well as empirical support in the favor of this relationship within the realm of music education. Moreover, the findings contribute to a finer-grain understanding of the mechanism through which SME influences EA by emphasizing the mediating role of SE. This aligns with SCT (Bandura, 1989), which posits that active engagement and observation of sustainable practices can lead to increased EA (Barrón et al., 2022). By empirically validating this theoretical framework within the context of music education, this study strengthens the theoretical underpinning of how education can drive environmental awareness and action.

Lastly, the study's fourth and fifth hypotheses postulating the interaction effect of IPA in the relationship between SME and SE as well as SME and EA through SE, are confirmed by the empirical analyses. These findings not only align with prior research but also shed light on the nuanced relationship between IPA, SE, and EA in the context of SME. For instance, our study supports the theoretical notion that IPA can play a pivotal role in strengthening the association between SME and SE. Previous research has emphasized the positive influence of IPA on SE (Alvarez-Bell et al., 2017; Gilboy et al., 2015; Herrington and Reeves, 2011). The theoretical implication engenders that when educators incorporate creative and dynamic teaching methods within SME, it enhances the learning experience, making it more engaging and immersive. Consequently, students become more actively involved in sustainability-themed music activities, fostering their SE. This finding underscores the importance of pedagogical innovation in music education. While previous research has explored the influence of innovative teaching methods on students' environmental behaviors (Jensen and Schnack, 2006), our findings suggest a deeper level of interconnectedness. Such that when IPA is effectively integrated into SME, it intensifies the link between SE and then EA. In this milieu, SE serves as a mediator through which IPA channel the positive impact of SME toward heightened environmental awareness. This theoretical insight emphasizes the importance of the method of delivery in sustainability education and suggests that innovative teaching methods can be instrumental in nurturing environmentally conscious individuals.

### Practical implications

The findings of this study carry several practical implications that can inform both music educators and policymakers. First and foremost, the author suggests that music educators consider integrating SME into their curricula, as these findings advocate for its potential to not only enhance SE but also to foster greater EA among students. Furthermore, the study recommends that institutions should explore IPA as a means to amplify the positive effects of SME on SE. This synergy between SME and IPA can create a rich and dynamic learning environment that encourages students to actively engage with sustainability principles.

Moreover, these findings highlight the potential of music education programs to contribute to broader environmental education efforts. Given the emotive power of music, the study recommends that educators and policymakers leverage music's capacity to elicit emotional responses to environmental issues. By doing so, we can inspire greater consciousness and action on ecological matters among students and the broader community. A key strategy to reinforce this connection is the adoption of eco-friendly music practices, such as promoting digital sheet music, using recycled or sustainably sourced materials for instruments, and integrating sustainability-themed compositions into student projects. These approaches not only reduce the environmental impact of music education but also create a framework through which students can actively engage in sustainable music production and performance.

Additionally, the author invites policymakers' attention to the significance of integrating sustainability themes within educational policy frameworks. Encouraging the inclusion of SME and IPA within music education guidelines can lead to more comprehensive and impactful sustainability education. Institutions can further strengthen this initiative by developing partnerships with environmental organizations, fostering community-based projects where students compose and perform music centered on sustainability, and organizing carbon-neutral music festivals or performances that align with eco-conscious principles. By nurturing environmentally conscious individuals through music education, we can contribute to a future generation that actively participates in addressing pressing global environmental challenges.

In summary, these findings advocate for the incorporation of SME and IPA in music education, highlighting their potential to enhance SE and foster greater EA. This study recommends a holistic approach to sustainability education, inviting both educators and policymakers to consider music's unique role in inspiring positive environmental action.

## Limitations and recommendations for future studies

As with any research endeavor, this study is not without its limitations, which in turn offer valuable avenues for future exploration. Firstly, it is essential to acknowledge that the findings are based on a specific context, in this case, music education in China. Thus, caution should be exercised when generalizing the results to different cultural and educational settings. Hence, future studies should encompass a broader range of cultural and geographical contexts to enhance the external validity of the findings.

Secondly, this research relied primarily on self-report measures through questionnaires, which can introduce response bias and social desirability effects. Incorporating multiple data sources, such as observations and interviews, could provide a more comprehensive understanding of the relationships under investigation.

Furthermore, the study's design is cross-sectional, limiting its ability to infer causality. Future research can employ longitudinal or experimental designs to establish causal relationships and explore the long-term effects of SME and IPA on SE and EA.

Additionally, the moderating role of IPA was examined but not thoroughly explored in terms of the specific types or qualities of IPA. Future studies can delve deeper into the nuances of IPA and its varying effects on the SME-SE-EA nexus.

Lastly, while the study focused on the educational aspects of SME, future research could extend its scope to include the assessment of practical implementations and outcomes of sustainability practices within music education programs.

## Data Availability

The raw data supporting the conclusions of this article will be made available by the authors, without undue reservation.
