# Supplementary material for: Unraveling the pathways of sustainable music education: a moderated mediation analysis of environmental awareness, pedagogical approaches, and student engagement
Source: Front Psychol. 2025 May 9;16:1554944. doi: 10.3389/fpsyg.2025.1554944 (PMC12098422; doi:10.3389/fpsyg.2025.1554944)
Supplement: Supplementary file 1 [file Data_Sheet_1.docx]

**Appendix-1 (Questionnaire)**

**Sustainable music education**

"My music education includes discussions about sustainability and its relevance to the music industry."

"I have opportunities to engage in sustainable music practices, such as using eco-friendly instruments."

"Sustainability is integrated into the curriculum of my music education program."

"I receive guidance on how to make sustainable choices in music production and performance."

**Environmental awareness**

"I always discuss about environmental problems with my friends."

"I do not use plastic bags to wrap things."

"I deliver information on the environment to my family members."

"I involve in the environmental awareness activities in school."

**Student engagement**

"In my studies, I feel like I am bursting with energy."

"When studying or attending classes I feel strong and vigorous."

"When I get up in the morning, I feel like going to class."

"I can continue for a very long time when I am studying."

"When I’m studying, I feel mentally strong."

"At my studies I always persevere, even when things do not go well."

"Time flies when I’m studying."

"When I am studying, I forget everything else around me."

"I feel happy when I am studying intensively."

"I am immersed in my studies."

"I can get carried away by my studies."

"It is difficult to detach myself from studying."

"I find my studies to be full of meaning and purpose."

"I am enthusiastic about my studies."

"My studies inspire me."

"I am proud of my studies."

"I find my studies challenging."

**Innovative pedagogical approaches**

"I have experienced innovative teaching methods that integrate sustainability concepts in my music education."

"The use of innovative pedagogical approaches in my music classes has made learning about environmental issues more engaging."

"Innovative pedagogical approaches, such as project-based learning or technology-enhanced lessons, have increased my interest in sustainability within music education."

"I feel that innovative teaching methods used in sustainability music education have enhanced my critical thinking skills."

"The innovative pedagogical approaches employed in my music classes have encouraged me to explore sustainability-related topics beyond the classroom."

"I believe that innovative teaching methods in sustainability music education have positively impacted my ability to connect music with environmental awareness."

"The innovative pedagogical approaches in sustainability music education have allowed me to apply sustainability principles practically in musical projects and performances."

"I find that innovative pedagogical approaches make sustainability-related content more accessible and relatable within my music education."

"The use of innovative pedagogical approaches in SME has motivated me to become more environmentally conscious in my musical endeavors."

"Innovative teaching methods have deepened my understanding of the relationship between music and sustainability."
